# Thiamine supplementation may be associated with improved prognosis in patients with sepsis

**DOI:** 10.1017/S0007114522003373

**Published:** 2023-07-28

**Authors:** Luming Zhang, Feng Zhang, Shaojin Li, Fengshuo Xu, Xiaoyu Zheng, Tao Huang, Jun Lyu, Haiyan Yin

**Affiliations:** 1 Department of Intensive Care Unit, The First Affiliated Hospital of Jinan University, Guangzhou, Guangdong Province 510630, People’s Republic of China; 2 Department of Clinical Research, The First Affiliated Hospital of Jinan University, Guangzhou, Guangdong Province, People’s Republic of China; 3 Department of Orthopaedics, The First Affiliated Hospital of Jinan University, Guangzhou, Guangdong Province, People’s Republic of China; 4 School of Public Health, Xi’an Jiaotong University Health Science Center, Xi’an, Shaanxi Province, People’s Republic of China

**Keywords:** Thiamine, Sepsis, Generalised boosted model, Inverse probability of treatment weighting, Doubly robust estimation

## Abstract

Sepsis is a clinical syndrome characterised by a severe disorder of pathophysiology caused by infection of pathogenic micro-organisms. The addition of antioxidant micronutrient therapies such as thiamine to sepsis treatment remains controversial. This study explored the effect of thiamine on the prognosis of patients with sepsis. This study was a retrospective study involving patients with sepsis from the Medical Information Mart for Intensive Care IV. Patients were divided into two groups, the thiamine received group (TR) and the thiamine unreceived group (TUR), according to whether they were supplemented with thiamin via intravenous while in the intensive care unit (ICU). The primary outcome was ICU mortality. The association between thiamine and outcome was analysed using the Cox proportional hazards regression model, propensity score matching (PSM), generalised boosted model-based inverse probability of treatment weighting (IPTW) and doubly robust estimation. A total of 11 553 sepsis patients were enrolled in this study. After controlling for potential confounders using Cox regression models, the TR group had a statistically significantly lower ICU mortality risk than the TUR group. The hazard ratio of ICU mortality for the TR group was 0·80 (95 % CI 0·70, 0·93). We obtained the same results after using PSM, IPTW and doubly robust estimation. Supplementation with thiamine has a beneficial effect on the prognosis of patients with sepsis. More randomised controlled trials are needed to confirm the effectiveness of thiamine supplementation in the treatment of sepsis.

Sepsis is a clinical syndrome characterised by a severe disorder of pathophysiology caused by infection of pathogenic micro-organisms. To facilitate early identification of this disease, Sepsis-3 defines it as life-threatening organ dysfunction resulting from a dys-regulated host response to infection. This means that the diagnosis is considered to be met when the patient has a suspected or confirmed infection combined with the Sequential Organ Failure Assessment ≥2^(1)^. Despite the lack of global epidemiological data on sepsis^(2)^, current studies estimate that it affects more than 30 million people annually and may cause 6 million deaths^(3)^, making it a major worldwide public health problem^(4)^. Currently, various antibiotics and organ support therapies are widely used in the clinical treatment of sepsis, but the mortality rate remains high^(5)^. The mechanism of sepsis is very complex, and further research is needed.

The primary causative factors of sepsis or the alterations in the internal environment and haemodynamics that occur during disease progression can lead to massive cytokine release, oxidative stress imbalance and mitochondrial dysfunction in the organism. When antioxidant defences are overwhelmed, reactive oxygen species cause cellular damage, leading to organ dysfunction and tissues becoming hypoxic, along with an increase in free radical production and oxidative stress. Mitochondria are both sites of reactive oxygen species production and targets of reactive oxygen species-mediated damage, therefore playing a prominent role in the pathogenesis of sepsis^(6,7)^. For example, it was found that mitochondrial dysfunction plays a potential role in sepsis-induced acute kidney injury (AKI)^(8)^. Linear stereoscopic dysfunction and biological failure are also important causes of cardiac insufficiency in patients with sepsis^(9)^. The restoration of mitochondrial function, also known as metabolic resuscitation, may help relieve organ dysfunction in septic patients and improve their prognosis. Thiamine (vitamin B_1_) is a water-soluble vitamin that plays an essential role in mitochondrial energy metabolism and is involved in multiple cellular metabolic processes in mitochondria and peroxisomes in the body. Moreover, it provides a certain resistance to oxidative stress of cells^(10)^. Thiamine deficiency occurs in most acute and critically ill patients and may be related to the accumulation of lactate^(11)^. A retrospective study revealed that in the early stages of sepsis, intravenous vitamin C along with corticosteroids and thiamine can reduce patient mortality and prevent organ failure^(12)^. A randomised, double-blind, controlled study showed that intravenous thiamine administration reduces lactate production in a thiamine-deficient subgroup of patients with septic shock^(13)^. Thiamine supplementation has also been shown to reduce the risk of sepsis-related kidney injury^(14)^.

Mitochondrial dysfunction is commonly observed in critically ill patients, especially in those experiencing sepsis. The addition of antioxidant micronutrient therapies such as ascorbic acid and thiamine to sepsis treatment remains controversial^(15,16)^. Here, we conducted a retrospective cohort study based on the Medical Information Mart for Intensive Care IV, a sizeable critical disease database, to investigate the effect of thiamine on the prognosis of patients with sepsis.

## Methods

### Data source

The Medical Information Mart for Intensive Care (MIMIC) IV^(17)^ is a free open-access database^(18)^ containing comprehensive information on tens of thousands of critically ill patients from 2008 to 2019, including comprehensive information on the patient’s hospital period, laboratory tests, medication and vital signs. The data used in this study were from MIMIC-IV version 0.4. The database was approved by the Massachusetts Institute of Technology and Beth Israel Deaconess Medical Center, and consent was obtained for the original data collection. Researchers need to complete the corresponding courses and obtain certificates before they can access and carry out data extraction (Record ID: 38601114). Considering that the database hides information about the identity of the patient, informed consent is not required.

### Population

Patients who met the diagnosis of Sepsis-3 in the Medical Information Mart for Intensive Care IV database were included, whereas patients who died within 24 h after admission to the intensive care unit (ICU) and patients younger than 18 years were excluded.

### Data extraction

Structured query language was used to extract data from the database. The data collected included age, sex, weight, ethnicity, first care unit, the severity of the disease (assessed using Sequential Organ Failure Assessment and Acute Physiological Score III), ventilator use, vasopressor use, continuous renal replacement therapy use, Charlson co-morbidity index and co-morbidities including myocardial infarction, congestive heart failure, peripheral vascular disease, cerebrovascular disease, dementia, chronic pulmonary disease, rheumatic disease, peptic ulcer disease, mild liver disease, severe liver disease, diabetes uncomplicated, diabetes complicated, paraplegia, renal disease, malignant cancer, metastatic solid tumour and AIDS. Results of the first laboratory examination after admission to the ICU included leucocyte count, Hb, platelet count, lactate, creatinine, urea nitrogen, glucose, PaCO_2_, pH and PaO_2_. The mean values of vital signs within 24 h of ICU admission include heart rate, mean arterial pressure, respiratory rate, temperature, pulse oximetry-derived oxygen saturation (SpO_2_) and total urine output in the first 24 h.

The primary outcome included ICU mortality, while the secondary outcomes included 28-d mortality, ventilation-free days in 28 d, vasopressor-free days in 28 d and incidence of AKI within 7 d after diagnosis of sepsis.

### Statistical analyses

The study population was divided into two groups, the thiamine received group (TR) and the thiamine unreceived group (TUR), based on whether thiamin was supplemented intravenously while in the ICU.

Missing data in public databases is a common phenomenon. In this study, we only included variables with missing rates less than 20 %, and the missing parts were filled using multiple imputation. This function can be implemented by the ‘mice’ package of the R programme. Data for continuous variables were expressed as median (interquartile range), and categorical data were presented as frequency. Mann–Whitney U test was used for continuous variables, and the *χ* ^2^ test or Fisher’s exact test was used for categorical variables. The relationship between thiamine and ICU mortality was measured using Cox proportional risk models, with estimated hazard ratios (HR) and 95 % CI. The effects of thiamine on the ventilation-free days and vasopressor-free days in 28 d were analysed by linear regression, expressed as regression coefficients (Coefs) with 95 % CI. The association of thiamine with the occurrence of AKI after sepsis diagnosis was performed by logistic regression analysis and rendered as OR with 95 % CI. Multivariate analysis was used to control for confounders. Confounders included age, sex, weight, ethnicity, first care unit, Sequential Organ Failure Assessment, Acute Physiological Score III, ventilator use, vasopressor use, continuous renal replacement therapy use, Charlson co-morbidity index, myocardial infarct, congestive heart failure, peripheral vascular disease, cerebrovascular disease, dementia, chronic pulmonary disease, rheumatic disease, peptic ulcer disease, mild liver disease, severe liver disease, diabetes uncomplicated, diabetes complicated, paraplegia, renal disease, malignant cancer, metastatic solid tumour, AIDS, leucocyte, Hb, platelet, lactate, creatinine, urea nitrogen, glucose, PaCO_2_, pH, PaO_2,_ heart rate, mean arterial pressure, respiratory rate, temperature, SpO_2_ and urine output.

Propensity score matching (PSM) and inverse probability of treatment weighting methods were conducted to balance the baseline characteristics of the two groups of patients. Standard mean differences are commonly used to make quantitative comparisons between the mean (continuous covariate) or rate (categorical covariate) of covariates dealing with groups. Some researchers suggest that the baseline covariate is considered to have reached an acceptable equilibrium level when the normalisation difference is less than 0·2^(19,20)^. In this study, PSM applied one-to-one matching of the nearest neighbours, while inverse probability of treatment weighting used the generalised boosted model^(21)^ to estimate the necessary tendency of the weighted score. The generalised boosted model estimation method estimates the use of a flexible dual propensity score for the treatment of indicators. It involves an iterative process with multiple regression trees to capture the treatment allocation and complex nonlinear relationship between pretreatment covariates without excessive fitting data. Studies have shown that among various propensity score estimation methods, generalised boosted model provides the estimated weight, which achieves the best balance among processing variables^(22)^. When imbalance still exists after weighting, the doubly robust estimation combines the multivariate regression model with the IPTW model, which can be used to eliminate residual confounding factors^(23)^. This results in more accurate estimates of therapeutic effects^(24)^.

A two-sided *P* < 0·05 was considered statistically significant. R (version 4.0.3) was used for all statistical analyses.

## Results

### Subject characteristics

**Fig. 1.**
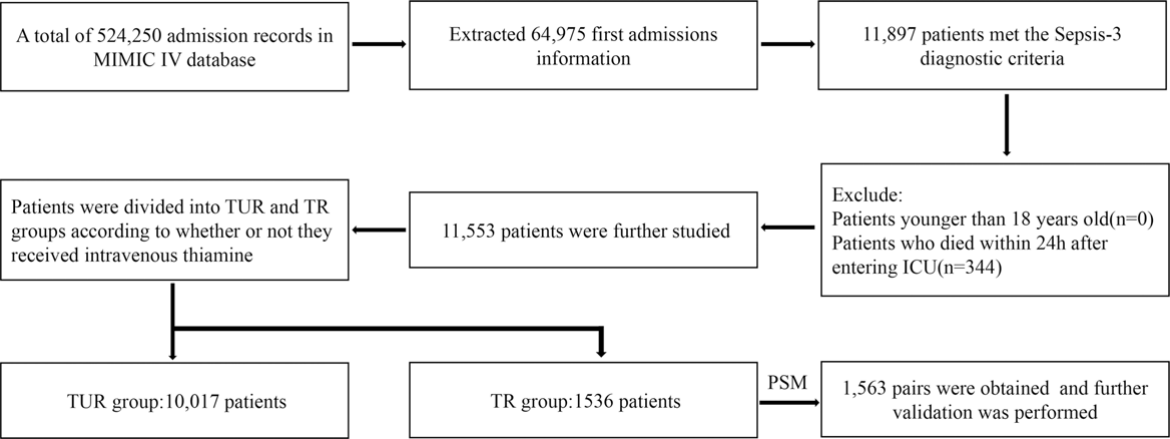
Flow chart of study cohort selection.

A total of 11 553 patients meeting the selection criteria were included in this study (Fig. 1). The patients with sepsis were divided into two groups according to whether they received intravenous thiamine or not. The TUR group included 10 017 patients and the TR group included 1536 patients. Table 1 summarises the characteristics of the patients, including general conditions, degree of illness, co-morbidities and laboratory parameters. In the original population, the age of the TR group was 59 (49, 68), which is lower than that of the TUR group 68 (56, 79). The Acute Physiological Score III (65 (49, 89)) and Sequential Organ Failure Assessment score (3 (2, 5)) in the TR group were higher than those in the TUR group (54 (41, 72) and 3 (2, 4), respectively). The length of ICU stay in the TR group was longer than that in the TUR group (8·48 (3·76, 15·88) *v*. 4·15 (2·02, 9·61)).

**Table 1.** Baseline characteristics of the study population (median (interquartile range [IQR]); numbers and percentages)

Characteristic	Original population				*P*	Matched population				*P*
	TUR		TR			TUR		TR		
	median	IQR	median	IQR		median	IQR	median	IQR	
*n*	10 017		1536			1536		1536		
Age (year)	68·00	56·00, 79·00	59·00	49·00, 68·00	<0·001	59·00	48·00, 71·00	59·00	49·00, 68·00	0·471

	*n*	%	*n*	%		*n*	%	*n*	%	
Sex					<0·001					0·714
Male	5139	51·3	901	58·7		890	57·9	901	58·7	
Female	4878	48·7	635	41·3		646	42·1	635	41·3	

	OR	95 % CI	OR	95 % CI		OR	95 % CI	OR	95 % CI	
Weight (kg)	77·50	65·00, 94·30	78·90	65·50, 96·30	0·103	79·00	66·40, 96·16	78·70	65·57, 96·15	0·635

	*n*	%	*n*	%		*n*	%	*n*	%	
Ethnicity					<0·001					0·931
White	6801	67·9	949	61·8		959	62·4	949	61·8	
Black	1134	11·3	175	11·4		171	11·1	175	11·4	
Others	2082	20·8	412	26·8		406	26·4	412	26·8	
First care unit					<0·001					<0·001
MICU/SICU/TSICU	7753	77·4	1322	86·1		1297	84·4	1322	86·1	
CCU	1934	19·3	156	10·2		215	14·0	156	10·2	
Others	330	3·3	58	3·8		24	1·6	58	3·8	
Severe score										

	OR	95 % CI	OR	95 % CI		OR	95 % CI	OR	95 % CI	
SOFA	3·00	2·00, 4·00	3·00	2·00, 5·00	<0·001	3·00	2·00, 5·00	3·00	2·00, 5·00	0·340
APS III	54·00	41·00, 72·00	65·00	49·00, 89·00	<0·001	66·50	48·00, 88·00	65·00	49·00, 89·00	0·634

	*n*	%	*n*	%		*n*	%	*n*	%	
Ventilator					<0·001					0·424
No	3448	34·4	311	20·2		330	21·5	311	20·2	
Yes	6569	65·6	1225	79·8		1206	78·5	1225	79·8	
Vasopressor					<0·001					0·913
No	5833	58·2	645	42·0		641	41·7	645	42·0	
Yes	4184	41·8	891	58·0		895	58·3	891	58·0	
CRRT					<0·001					0·357
No	9336	93·2	1303	84·8		1322	86·1	1303	84·8	
Yes	681	6·8	233	15·2		214	13·9	233	15·2	

	OR	95 % CI	OR	95 % CI		OR	95 % CI	OR	95 % CI	
Charlson co-morbidity index	3·00	1·00, 5·00	3·00	1·00, 4·00	0·486	3·00	1·00, 4·00	3·00	1·00, 4·00	0·111
Co-morbidities										

	*n*	%	*n*	%		*n*	%	*n*	%	
Myocardial infarct					0·128					0·678
No	8420	84·1	1315	85·6		1324	86·2	1315	85·6	
Yes	1597	15·9	221	14·4		212	13·8	221	14·4	
Congestive heart failure					<0·001					1·000
No	6732	67·2	1165	75·8		1165	75·8	1165	75·8	
Yes	3285	32·8	371	24·2		371	24·2	371	24·2	
Peripheral vascular disease					<0·001					0·502
No	8834	88·2	1410	91·8		1421	92·5	1410	91·8	
Yes	1183	11·8	126	8·2		115	7·5	126	8·2	
Cerebrovascular disease					1·000					0·650
No	8538	85·2	1309	85·2		1299	84·6	1309	85·2	
Yes	1479	14·8	227	14·8		237	15·4	227	14·8	
Dementia					0·005					0·524
No	9455	94·4	1477	96·2		1469	95·6	1477	96·2	
Yes	562	5·6	59	3·8		67	4·4	59	3·8	
Chronic pulmonary disease					0·125					0·672
No	7396	73·8	1163	75·7		1174	76·4	1163	75·7	
Yes	2621	26·2	373	24·3		362	23·6	373	24·3	
Rheumatic disease					0·087					1·000
No	9638	96·2	1492	97·1		1493	97·2	1492	97·1	
Yes	379	3·8	44	2·9		43	2·8	44	2·9	
Peptic ulcer disease					<0·001					0·366
No	9727	97·1	1452	94·5		1464	95·3	1452	94·5	
Yes	290	2·9	84	5·5		72	4·7	84	5·5	
Mild liver disease					<0·001					0·619
No	8665	86·5	1017	66·2		1031	67·1	1017	66·2	
Yes	1352	13·5	519	33·8		505	32·9	519	33·8	
Severe liver disease					<0·001					0·432
No	9341	93·3	1239	80·7		1257	81·8	1239	80·7	
Yes	676	6·7	297	19·3		279	18·2	297	19·3	
Diabetes uncomplicated					<0·001					1·000
No	7471	74·6	1218	79·3		1219	79·4	1218	79·3	
Yes	2546	25·4	318	20·7		317	20·6	318	20·7	
Diabetes complicated					<0·001					0·692
No	8816	88·0	1408	91·7		1415	92·1	1408	91·7	
Yes	1201	12·0	128	8·3		121	7·9	128	8·3	
Paraplegia					0·609					0·937
No	9426	94·1	1451	94·5		1449	94·3	1451	94·5	
Yes	591	5·9	85	5·5		87	5·7	85	5·5	
Renal disease					<0·001					0·377
No	7235	72·2	1279	83·3		1298	84·5	1279	83·3	
Yes	2782	27·8	257	16·7		238	15·5	257	16·7	
Malignant cancer					0·032					0·571
No	8708	86·9	1366	88·9		1355	88·2	1366	88·9	
Yes	1309	13·1	170	11·1		181	11·8	170	11·1	
Metastatic solid tumour					0·094					0·797
No	9459	94·4	1467	95·5		1463	95·2	1467	95·5	
Yes	558	5·6	69	4·5		73	4·8	69	4·5	
AIDS					0·042					0·253
No	9926	99·1	1513	98·5		1521	99·0	1513	98·5	
Yes	91	0·9	23	1·5		15	1·0	23	1·5	
Laboratory tests										

	OR	95 % CI	OR	95 % CI		OR	95 % CI	OR	95 % CI	
Leucocytes (K/µl)	11·60	7·90, 16·60	11·50	7·20, 16·90	0·217	11·60	7·50, 16·70	11·50	7·20, 16·90	0·643
Hb (g/dl)	10·00	8·70, 11·50	10·10	8·60, 11·90	0·020	10·20	8·70, 11·80	10·10	8·60, 11·90	0·661
Platelet (K/µl)	190·00	129·00, 266·00	164·00	96·50, 239·00	<0·001	165·00	100·00, 238·25	164·00	96·75, 239·00	0·550
Lactate (mmol/l)	1·70	1·20, 2·60	2·10	1·30, 3·60	<0·001	2·10	1·40, 3·50	2·00	1·30, 3·52	0·447
Creatinine (mg/dl)	1·20	0·80, 1·90	1·10	0·70, 2·00	0·061	1·10	0·80, 1·80	1·10	0·70, 2·00	0·491
Urea nitrogen (mg/dl)	25·00	16·00, 43·00	22·00	13·00, 39·75	<0·001	23·00	15·00, 39·00	22·00	13·00, 39·25	0·072
Glucose (mg/dl)	130·00	104·00, 172·00	126·00	101·00, 170·00	0·007	128·00	102·00, 170·25	126·00	101·00, 170·00	0·233
PaCO_2_ (mmHg)	41·00	35·00, 48·00	39·00	33·00, 46·00	<0·001	39·00	33·00, 45·00	39·00	33·00, 46·00	0·546
pH	7·37	7·31, 7·43	7·36	7·28, 7·42	<0·001	7·36	7·29, 7·42	7·36	7·28, 7·42	0·486
PaO_2_ (mmHg)	91·00	54·00, 167·00	83·00	49·25, 147·75	<0·001	86·00	51·00, 147·00	82·00	48·75, 145·00	0·221
Vital signs										
Heart rate (beats/min)	86·92	75·91, 98·96	93·11	81·11, 105·95	<0·001	93·55	81·34, 106·45	93·11	81·11, 105·95	0·651
Mean arterial pressure (mmHg)	73·80	68·29, 80·90	75·55	69·62, 83·08	<0·001	75·58	70·12, 83·62	75·55	69·62, 83·08	0·408
Respiratory rate (breaths/min)	19·60	17·20, 22·50	20·29	17·50, 23·62	<0·001	20·13	17·61, 23·57	20·30	17·50, 23·61	0·715
Temperature (°C)	36·87	36·59, 37·22	36·93	36·63, 37·37	<0·001	36·94	36·63, 37·38	36·93	36·63, 37·37	0·626
SpO_2_ (%)	97·17	95·78, 98·50	97·24	95·76, 98·63	0·365	97·22	95·71, 98·60	97·24	95·76, 98·63	0·813
Urine output (ml)	1380·00	814·00, 2172·75	1300·00	695·00, 2200·00	0·002	1280·00	672·25, 2194·25	1280·00	671·50, 2165·00	0·834
Outcomes										

	*n*	%	*n*	%		*n*	%	*n*	%	
ICU mortality					<0·001					1·000
No	8788	87·7	1236	80·5		1237	80·5	1236	80·5	
Yes	1229	12·3	300	19·5		299	19·5	300	19·5	

	OR	95 % CI	OR	95 % CI		OR	95 % CI	OR	95 % CI	
Length of ICU stay (days)	4·15	2·02, 9·61	8·48	3·76, 15·88	<0·001	6·56	2·85, 13·26	8·48	3·76, 15·88	<0·001

TR, thiamine received group; TUR, thiamine unreceived group; PSM, propensity score matching; MICU, medical intensive care unit; SICU, surgical intensive care unit; TSICU, trauma surgical intensive care unit; CCU, coronary care unit; SOFA, Sequential Organ Failure Assessment; APS III, Acute Physiological Score III; CRRT, continuous renal replacement therapy; SpO_2_, pulse oximetry-derived oxygen saturation.

### Cox proportional hazards regression model

After adjusting for confounders, the TR group showed a reduced risk of all-cause sepsis mortality rates. In comparison with the TUR group, the TR group had a statistically significantly lower risk of ICU mortality. The HR of ICU mortality for the TR group was 0·80 (95 % CI 0·70, 0·93), indicating that, compared with the TUR group, the TR group had 0·80 times the risk for ICU mortality (Table 2).

**Table 2.** Analysis of the associations between outcomes and thiamine received (Hazard ratios; odds ratios; 95 % confidence intervals)

	HR*/OR†/Coef‡	95 % CI	*P*
Primary outcome			
ICU mortality			
Multivariate model	0·80	0·70, 0·93*	0·002
PSM	0·82	0·70, 0·96*	0·015
IPTW	0·80	0·69, 0·94*	0·005
Doubly robust with unbalanced covariates§	0·80	0·69, 0·94*	0·005
Doubly robust with all covariates	0·82	0·71, 0·96*	0·013
Secondary outcomes			
28-d mortality	0·82	0·71, 0·94*	0·004
Ventilation-free days in 28 d	1·09	0·52, 1·67‡	<0·001
Vasopressor-free days in 28 d	1·61	1·10, 2·12‡	<0·001
AKI after diagnosis of sepsis	1·01	1·67, 0·82†	0·928

PSM, propensity score matching; IPTW, inverse probability of treatment weighting.

* Cox proportional hazards regression models were used to calculate hazard ratios (HR) with 95 % CI.

† Logistic regression models were used to calculate OR with 95 % (CI).

‡ Linear regression models were used to calculate coefficients (Coefs) with 95 % (CI).

§ Confounders for the doubly robust with unbalanced covariates included age, sex, ventilator use, vasopressor use, CRRT use, first care unit, APS III, myocardial infarct, mild liver disease, severe liver disease, PaCO_2_, pH, glucose, platelet and SpO_2_. Confounders of other models are consistent with multivariate analysis.

### Propensity score matching and inverse probability of treatment weighting

To reduce confounding bias, we performed PSM based on whether thiamine was used. A total of 1563 pairs were successfully matched (online Supplementary Fig. S1). After matching, the imbalance was controlled based on a comparison of the patients’ clinical data (standard mean difference < 0·2, Table 1, online Supplementary Fig. S2). After inverse probability of treatment weighting, the standard mean difference and the baseline characteristics between the two groups of the virtual population can be seen in online Supplementary Table S1.

Although the baseline differences between the two groups were well controlled, there were still variables that were statistically different. We separately included variables with differences in the matched and weighted populations in the Cox regression models, yielding results with consistent trends in the original population. And by including all of the above variables in the multivariate Cox regression models, the results remained stable (Table 2).

### Secondary outcome studies with the whole population

Based on the original population, we conducted a series of secondary outcome analyses to confirm the benefits of thiamine on the prognosis of patients. Multivariate Cox regression showed that the 28-d mortality of the TR group was improved (HR: 0·82; 95 % CI (0·71, 0·94)). The ventilation-free days in 28 d of the TR group were 1·09 d longer than those in the TUR group (Coef: 1·09; 95 % CI (0·52, 1·67)), and vasopressor-free days in 28 d of the TR group were 1·61 d longer than those in the TUR group (Coef: 1·61; 95 % CI (1·10, 2·12)). There was no significant difference in the probability of AKI after the diagnosis of sepsis between the two groups (Table 2).

### Subgroup analyses

**Fig. 2.**
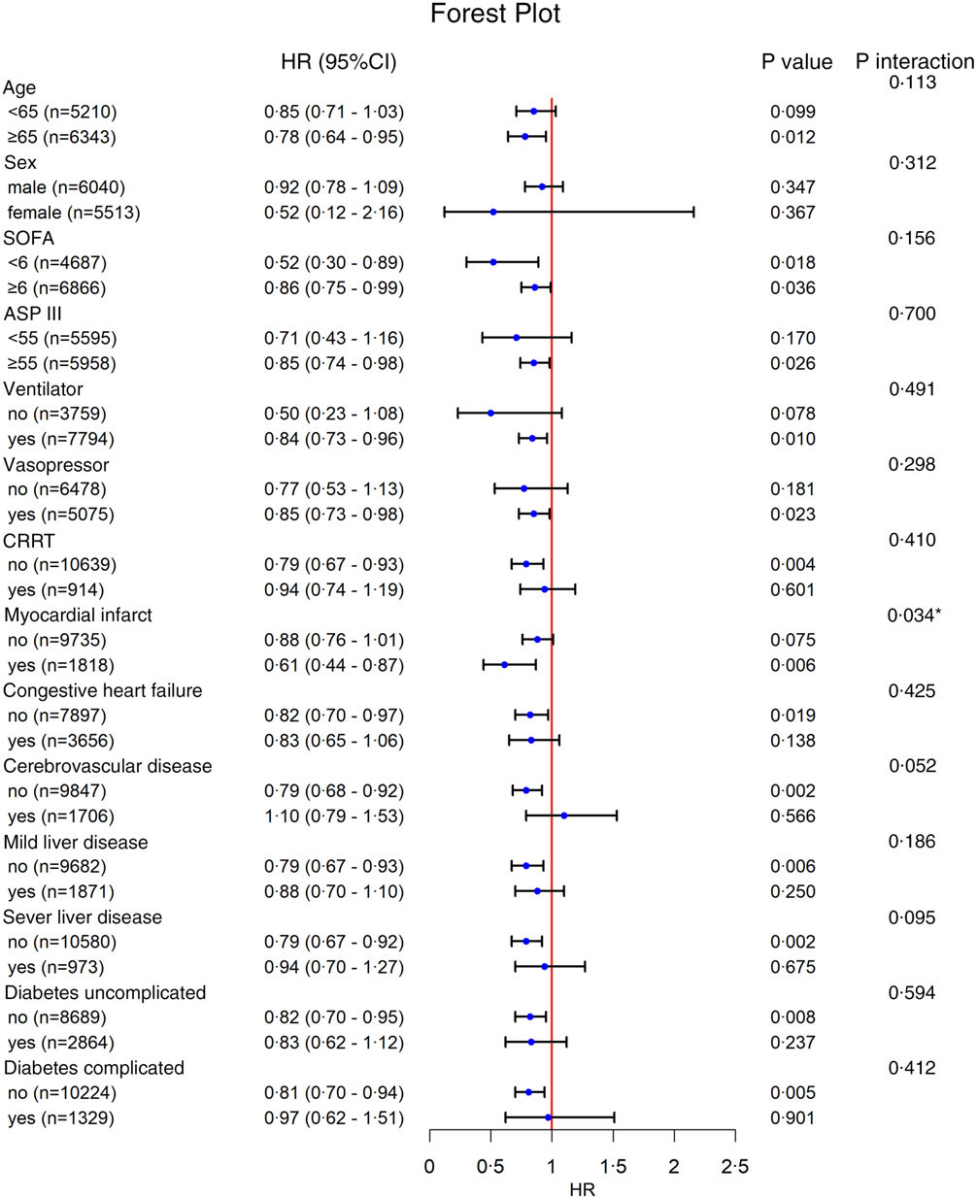
Subgroup analysis of the associations between ICU all-cause mortality and thiamine received, *P < 0·05. Confounders were consistent with the multivariate model in Table 2.

We performed a subgroup analysis of the primary outcomes by using clinically significant scores and several complications (Fig. 2, online Supplementary Table S2). A significant interaction was observed in MI complication. The HR of people without MI was 0·88 (95 % CI 0·76, 1·01), and the HR of people with MI was 0·61 (95 % CI 0·44, 0·87). The use of thiamine has a significant protective effect on patients with MI.

## Discussion

In the present study, we investigated the link between thiamine supplementation and sepsis mortality. After controlling for potential confounders using Cox regression models, we found that the use of thiamine is associated with the improvement of ICU all-cause mortality in patients with sepsis. This observation was also supported by the double robustness validation on the matched and weighted populations. Secondary outcome analyses demonstrated that the use of thiamine was associated with the improvement of 28-d mortality, ventilation-free days in 28 d and vasopressor-free days in 28 d in patients with sepsis, while there was no obvious association with the occurrence of AKI.

The biologically active form of thiamine, thiamine pyrophosphate, is an essential co-enzyme for glucose metabolism, which provides energy through the regulation of mitochondrial production of NADPH and ATP^(25)^. Additionally, thiamine levels are positively correlated with the activity of glutathione peroxidase, a major component of the cellular antioxidant system, which is also critical in scavenging oxygen-free radicals^(26)^. Thiamine’s important role in cellular energy metabolism, oxidative stress and maintenance of mitochondrial function provides the basis for its therapeutic application in sepsis. The human body cannot synthesise thiamine by itself and has limited storage, so it must rely on external sources to avoid deficiency. Patients with sepsis have an increased need for thiamine due to infection, but the accompanying decrease in nutritional intake makes its deficiency common^(27)^. Moreover, the combination of multi-organ dysfunction in sepsis patients may impede the transport and utilisation of thiamine, and operations such as haemodialysis may also increase its excretion^(28)^. Animal tests show that sepsis inhibits the intestinal absorption of thiamine, resulting in less ATP production and affecting energy metabolism^(29)^. In one study, the prevalence of thiamine deficiency in patients with sepsis was 10 % upon initial admission, increasing to 20 % within the next 72 h^(30)^. Thiamine deficiency is common in critically ill adults and children^(26,31)^. A retrospective analysis of critically ill patients showed that thiamine levels were significantly higher in patients who survived than in those who died, and thiamine deficiency is associated with higher mortality^(32)^, in agreement with our results. As such, thiamine supplementation is of great interest to improve the prognosis of patients. Although intravenous thiamine supplementation showed no statistically significant relationship with the occurrence of AKI after sepsis, it does reduce the time that patients are treated with a ventilator and vasopressors, which improves cardiopulmonary function and general condition. The effects of hypoxia, hormones and inflammatory mediators on mitochondria in critical conditions are thought to be functional changes rather than structural changes and may be reversible^(33)^. Therefore, exogenous supplementation with thiamine could promote the recovery of mitochondrial function, prevent progression of the disease and reduce the mortality of patients with sepsis.

Subgroup analysis showed a beneficial effect of thiamine in patients with MI, with an HR of 0·61 (95 % CI 0·44, 0·87). The cardiovascular system is essential for maintaining adequate organ perfusion, and its failure to function can affect the progression of sepsis. Cardiac muscle is rich in mitochondria that enable it to provide a large amount of energy for the systolic activity of the heart^(34)^. In patients with MI, the presence of necrotic and apoptotic myocardial cells leads to a reduction in the number of mitochondria, despite the ability of the rest of the healthy cells to compensate for the energy needed for myocardial activity. Sepsis under autonomic nervous system disorders, reduced blood volume and the release of endogenous cytokines all damage cardiac muscle^(35,36)^. In addition, the electron transport chain^(37)^, abnormal oxidative phosphorylation and various mechanisms mentioned above in sepsis can impair mitochondrial function^(38)^. Thus, intravenous thiamine supplementation may protect mitochondrial function in patients with MI, thereby reducing their risk of death.

### Strengths and limitations of the study

The advantage of this study is the use of the Medical Information Mart for Intensive Care IV database, which has a large sample size and contains relatively recent data, thus providing strong evidence for our conclusion. For further verification, we obtained the same results after adjusting for baseline levels by using PSM and inverse probability of treatment weighting. However, this study also has some limitations. First, this study is a single-centre clinical study. Second, we included only patients who received thiamine intravenously in consideration of gastrointestinal dysfunction in critical conditions. The prognostic impact of different routes of administration on patients could be studied in the future. Third, we did not consider the effects of specific injection doses and timing. Fourth, because patients have a large number of missing values of lactate levels 24 h after the diagnosis of sepsis, we were unable to determine whether thiamine improves the prognosis of patients by reducing the concentration of lactate. This can be verified in future studies. One more point was the lack of test results for thiamine levels in patients in the database, so we could not take it into account in this study.

### Conclusion

Supplementation with thiamine has a beneficial effect on the prognosis of patients with sepsis. More randomised controlled trials are needed to confirm the effectiveness of thiamine supplementation in the treatment of sepsis.

## Supplementary material

For supplementary material accompanying this paper visit https://doi.org/10.1017/S0007114522003373. click here to view supplementary material
