# Supplementary material for: Thiamine supplementation may be associated with improved prognosis in patients with sepsis
Source: Br J Nutr. 2022 Oct 19;130(2):239–48. doi: 10.1017/S0007114522003373 (PMC10277660; doi:10.1017/S0007114522003373)
Supplement: Supplementary file 1 [file S0007114522003373sup001.docx]

TableS1 Baseline characteristics of the study population after IPTW.

| Characteristic | After IPTW | |  |  |
| --- | --- | --- | --- | --- |
|  | TUR | TR | P | SMD |
| n | 11429.9 | 9285.4 |  |  |
| Age(year) | 65.44 (15.83) | 64.23 (15.26) | 0.024 | 0.078 |
| Gender (%) |  |  | 0.002 | 0.107 |
| male | 5945.9 (52.0) | 5324.4 (57.3) |  |  |
| female | 5484.0 (48.0) | 3961.0 (42.7) |  |  |
| Weight(kg) | 82.16 (26.45) | 81.33 (25.53) | 0.336 | 0.032 |
| Ethnicity (%) |  |  | 0.263 | 0.054 |
| White | 7705.5 (67.4) | 6029.7 (64.9) |  |  |
| Black | 1292.2 (11.3) | 1090.3 (11.7) |  |  |
| Others | 2432.2 (21.3) | 2165.4 (23.3) |  |  |
| First care unit (%) |  |  | 0.002 | 0.131 |
| MICU/SICU/TSICU | 8951.4 (78.3) | 7660.0 (82.5) |  |  |
| CCU | 2104.2 (18.4) | 1269.1 (13.7) |  |  |
| Others | 374.3 ( 3.3) | 356.4 ( 3.8) |  |  |
| Severe Score |  |  |  |  |
| SOFA | 3.56 (1.86) | 3.64 (1.95) | 0.131 | 0.045 |
| SAPS III | 59.87 (24.91) | 62.87 (25.30) | <0.001 | 0.120 |
| Ventilator (%) |  |  | 0.037 | 0.079 |
| no | 3767.8 (33.0) | 2722.2 (29.3) |  |  |
| yes | 7662.1 (67.0) | 6563.2 (70.7) |  |  |
| Vasopressor (%) |  |  | 0.001 | 0.117 |
| no | 6458.1 (56.5) | 4703.4 (50.7) |  |  |
| yes | 4971.8 (43.5) | 4582.0 (49.3) |  |  |
| CRRT (%) |  |  | 0.004 | 0.082 |
| no | 10563.2 (92.4) | 8366.0 (90.1) |  |  |
| yes | 866.7 ( 7.6) | 919.4 ( 9.9) |  |  |
| Charlson comorbidity index | 3.11 (2.57) | 3.07 (2.44) | 0.653 | 0.015 |
| Comorbidities |  |  |  |  |
| myocardial infarct (%) |  |  | 0.035 | 0.076 |
| no | 9656.0 (84.5) | 7580.2 (81.6) |  |  |
| yes | 1773.9 (15.5) | 1705.2 (18.4) |  |  |
| congestive heart failure (%) |  |  | 0.360 | 0.033 |
| no | 7789.6 (68.2) | 6469.7 (69.7) |  |  |
| yes | 3640.3 (31.8) | 2815.7 (30.3) |  |  |
| peripheral vascular disease (%) |  |  | 0.257 | 0.042 |
| no | 10112.0 (88.5) | 8336.5 (89.8) |  |  |
| yes | 1317.9 (11.5) | 948.9 (10.2) |  |  |
| cerebrovascular disease (%) |  |  | 0.447 | 0.026 |
| no | 9755.1 (85.3) | 7837.7 (84.4) |  |  |
| yes | 1674.8 (14.7) | 1447.7 (15.6) |  |  |
| dementia (%) |  |  | 0.936 | 0.003 |
| no | 10823.3 (94.7) | 8786.7 (94.6) |  |  |
| yes | 606.6 ( 5.3) | 498.8 ( 5.4) |  |  |
| chronic pulmonary disease (%) |  |  | 0.618 | 0.017 |
| no | 8484.2 (74.2) | 6962.2 (75.0) |  |  |
| yes | 2945.7 (25.8) | 2323.2 (25.0) |  |  |
| rheumatic disease (%) |  |  | 0.572 | 0.022 |
| no | 11004.1 (96.3) | 8977.0 (96.7) |  |  |
| yes | 425.8 ( 3.7) | 308.5 ( 3.3) |  |  |
| peptic ulcer disease (%) |  |  | 0.397 | 0.023 |
| no | 11080.0 (96.9) | 8963.5 (96.5) |  |  |
| yes | 349.9 ( 3.1) | 321.9 ( 3.5) |  |  |
| mild liver disease (%) |  |  | 0.009 | 0.069 |
| no | 9638.7 (84.3) | 7589.0 (81.7) |  |  |
| yes | 1791.2 (15.7) | 1696.4 (18.3) |  |  |
| severe liver disease (%) |  |  | 0.048 | 0.047 |
| no | 10510.0 (92.0) | 8414.4 (90.6) |  |  |
| yes | 919.9 ( 8.0) | 871.1 ( 9.4) |  |  |
| diabetes uncomplicated (%) |  |  | 0.176 | 0.048 |
| no | 8563.0 (74.9) | 7145.9 (77.0) |  |  |
| yes | 2866.9 (25.1) | 2139.5 (23.0) |  |  |
| diabetes complicated (%) |  |  | 0.559 | 0.021 |
| no | 10102.6 (88.4) | 8269.5 (89.1) |  |  |
| yes | 1327.3 (11.6) | 1015.9 (10.9) |  |  |
| paraplegia (%) |  |  | 0.970 | 0.001 |
| no | 10748.8 (94.0) | 8729.1 (94.0) |  |  |
| yes | 681.1 ( 6.0) | 556.4 ( 6.0) |  |  |
| renal disease (%) |  |  | 0.076 | 0.067 |
| no | 8379.0 (73.3) | 7076.3 (76.2) |  |  |
| yes | 3050.9 (26.7) | 2209.1 (23.8) |  |  |
| malignant cancer (%) |  |  | 0.682 | 0.014 |
| no | 9939.2 (87.0) | 8119.0 (87.4) |  |  |
| yes | 1490.7 (13.0) | 1166.4 (12.6) |  |  |
| metastatic solid tumor (%) |  |  | 0.965 | 0.002 |
| no | 10798.6 (94.5) | 8769.1 (94.4) |  |  |
| yes | 631.3 ( 5.5) | 516.4 ( 5.6) |  |  |
| aids (%) |  |  | 0.409 | 0.022 |
| no | 11320.9 (99.0) | 9176.2 (98.8) |  |  |
| yes | 109.0 ( 1.0) | 109.2 ( 1.2) |  |  |
| Laboratory tests |  |  |  |  |
| White blood cell (K/uL) | 13.52 (11.16) | 13.39 (9.67) | 0.688 | 0.013 |
| Hemoglobin (g/dl) | 10.18 (2.14) | 10.24 (2.19) | 0.410 | 0.027 |
| Platelet (K/uL) | 208.84 (123.29) | 198.41 (129.87) | 0.018 | 0.082 |
| Lactate (mmol/L) | 2.20 (1.70) | 2.28 (1.80) | 0.094 | 0.045 |
| Creatinine (mg/dl) | 1.74 (1.76) | 1.70 (1.57) | 0.358 | 0.029 |
| Urea nitrogen (mg/dl) | 33.39 (26.16) | 32.66 (26.24) | 0.410 | 0.028 |
| Glucose (mg/dl) | 152.17 (85.41) | 145.82 (75.96) | 0.007 | 0.079 |
| PaCO2 (mmhg) | 42.15 (12.70) | 41.17 (11.47) | 0.009 | 0.081 |
| pH | 7.36 (0.10) | 7.36 (0.10) | 0.038 | 0.063 |
| PaO2 (mmhg) | 123.30 (101.75) | 117.86 (96.83) | 0.105 | 0.055 |
| Vital signs |  |  |  |  |
| Heart rate (beats/min) | 88.52 (16.60) | 89.44 (16.45) | 0.086 | 0.055 |
| Mean arterial pressure (mmhg) | 75.42 (10.30) | 75.73 (10.53) | 0.361 | 0.030 |
| Respiratory rate (breaths/min) | 20.20 (4.11) | 20.30 (4.10) | 0.414 | 0.026 |
| Temperature (℃) | 36.90 (0.61) | 36.92 (0.59) | 0.510 | 0.020 |
| SpO2 (%) | 96.95 (2.14) | 97.10 (2.03) | 0.027 | 0.070 |
| urine output(ml) | 1624.73 (1231.89) | 1571.10 (1228.90) | 0.176 | 0.044 |
| Outcomes |  |  |  |  |
| ICU mortality |  |  | 0.109 | 0.048 |
| no | 9935.1 (86.9) | 7916.3 (85.3) |  |  |
| yes | 1494.8 (13.1) | 1369.1 (14.7) |  |  |
| Length of ICU stay (days) | 7.95 (11.29) | 10.44 (10.42) | <0.001 | 0.230 |

TableS2 Subgroup analysis of the associations between ICU all-cause mortality and thiamine received.

|  | TUR group | TR group |  |  |
| --- | --- | --- | --- | --- |
|  |  | HR(95%CI) | p-value | p-interaction |
| Age |  |  |  | 0.113 |
| <65(n=5210) | Reference | 0.85(0.71,1.03) | 0.099 |  |
| ≥65(n=6343) | Reference | 0.78(0.64,0.95) | 0.012 |  |
| Gender |  |  |  | 0.312 |
| male(n=6040) | Reference | 0.92(0.78,1.09) | 0.347 |  |
| female(n=5513) | Reference | 0.52(0.12,2.16) | 0.367 |  |
| SOFA |  |  |  | 0.156 |
| <6(n=4687) | Reference | 0.52(0.30,0.89) | 0.018 |  |
| ≥6(n=6866) | Reference | 0.86(0.75,0.99) | 0.036 |  |
| SASPIII |  |  |  | 0.700 |
| <55(n=5595) | Reference | 0.71(0.43,1.16) | 0.170 |  |
| ≥55(n=5958) | Reference | 0.85(0.74,0.98) | 0.026 |  |
| Ventilator |  |  |  | 0.491 |
| No(n=3759) | Reference | 0.50(0.23,1.08) | 0.078 |  |
| Yes(n=7794) | Reference | 0.84(0.73,0.96) | 0.010 |  |
| Vasopressor |  |  |  | 0.298 |
| No(n=6478) | Reference | 0.77(0.53,1.13) | 0.181 |  |
| Yes(n=5075) | Reference | 0.85(0.73,0.98) | 0.023 |  |
| CRRT |  |  |  | 0.410 |
| No(n=10639) | Reference | 0.79(0.67,0.93) | 0.004 |  |
| Yes(n=914) | Reference | 0.94(0.74,1.19) | 0.601 |  |
| Myocardial infarct |  |  |  | 0.034^*^ |
| No(n=9735) | Reference | 0.88(0.76,1.01) | 0.075 |  |
| Yes(n=1818) | Reference | 0.61(0.44,0.87) | 0.006 |  |
| Congestive heart failure |  |  |  | 0.425 |
| No(n=7897) | Reference | 0.82(0.70,0.97) | 0.019 |  |
| Yes(n=3656) | Reference | 0.83(0.65,1.06) | 0.138 |  |
| Cerebrovascular disease |  |  |  | 0.052 |
| No(n=9847) | Reference | 0.79(0.68,0.92) | 0.002 |  |
| Yes(n=1706) | Reference | 1.10(0.79,1.53) | 0.566 |  |
| Mild liver disease |  |  |  | 0.186 |
| No(n=9682) | Reference | 0.79(0.67,0.93) | 0.006 |  |
| Yes(n=1871) | Reference | 0.88(0.70,1.10) | 0.250 |  |
| Sever liver disease |  |  |  | 0.095 |
| No(n=10580) | Reference | 0.79(0.67,0.92) | 0.002 |  |
| Yes(n=973) | Reference | 0.94(0.70,1.27) | 0.675 |  |
| Diabetes uncomplicated |  |  |  | 0.594 |
| No(n=8689) | Reference | 0.82(0.70,0.95) | 0.008 |  |
| Yes(n=2864) | Reference | 0.83(0.62,1.12) | 0.237 |  |
| Diabetes complicated |  |  |  | 0.412 |
| No(n=10224) | Reference | 0.81(0.70,0.94) | 0.005 |  |
| Yes(n=1329) | Reference | 0.97(0.62,1.51) | 0.901 |  |


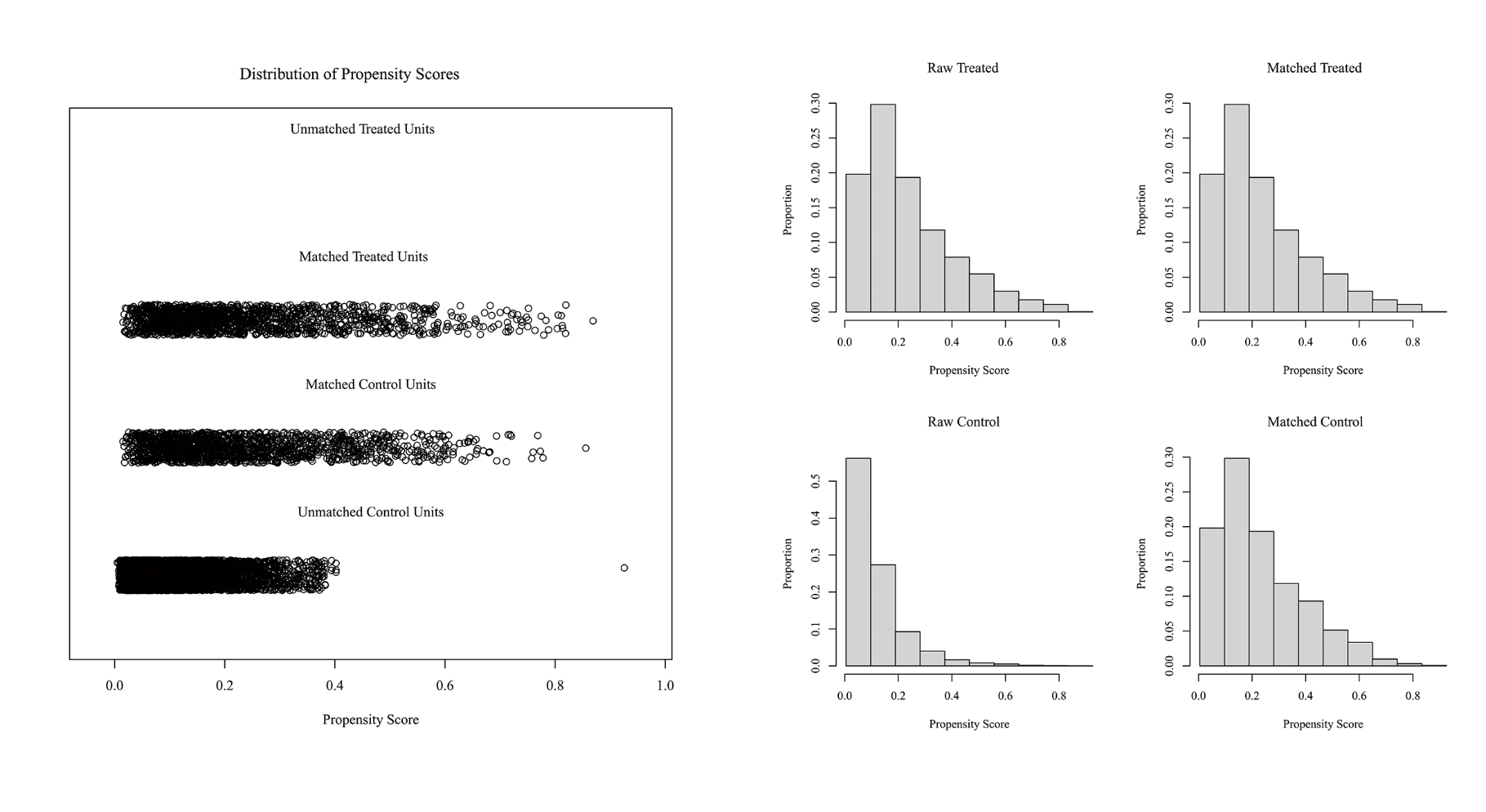


Figure S1 Distribution of 1:1 propensity Matching Score in Cluster(A) and Histogram(B).





Figure S2 SMD of covariable before and after PSM, IPTW.
